# The Same Instrument, Differently Tuned? Surveys, Delphi Studies, and the Search for Structured Disagreement

**DOI:** 10.1097/CCE.0000000000001457

**Published:** 2026-07-21

**Authors:** Prashant Nasa, Marcus J. Schultz, Denise Battaglini

**Affiliations:** 1 Department of Anaesthesia and Critical Care Medicine, The Royal Wolverhampton Trust, New Cross Hospital, Wolverhampton, United Kingdom.; 2 Department of Intensive Care Medicine, Amsterdam University Medical Centres, Amsterdam, The Netherlands.; 3 Department of Anesthesia, General Intensive Care and Pain Management, Division of Cardiothoracic and Vascular Anesthesia & Critical Care Medicine, Medical University of Vienna, Vienna, Austria.; 4 Department of Anaesthesiology, Rescue and Pain Medicine, Cantonal Hospital St. Gallen, HOCH Health Ostschweiz, St. Gallen, Switzerland.; 5 Nuffield Department of Medicine, University of Oxford, Oxford, United Kingdom.; 6 Mahidol Oxford Tropical Medicine Research Unit (MORU), Mahidol University, Bangkok, Thailand.; 7 Department of Surgical Sciences and Integrated Diagnostics (DISC), University of Genoa, Genoa, Italy.; 8 Department of Anesthesia and Intensive Care, IRCCS Azienda Ospedaliera Metropolitana, Genoa, Italy.

**Keywords:** cognitive biases, consensus, Delphi technique, implicit bias, methodology bias, survey research

Cross-sectional survey research and Delphi studies are important methodological tools in critical care research, and both have grown substantially in the past decade. Yet increased use has not been matched by increased rigor. Cross-sectional surveys are often distributed without adequate attention to question framing, sampling, or bias mitigation. Delphi studies frequently lack transparent reporting of panel composition, reasons for panelists attrition, and methodological biases during questionnaire development. The result, in both cases, can be conclusions that are incomplete, misleading, or simply wrong. And in the Delphi context, a false consensus that carries normative weight it has not earned. In this issue of *Critical Care Explorations*, Ramadurai et al (1) provide a timely and practical framework for survey design in critical care and draw parallels to the design of Delphi questionnaires. This prompts a deeper question: are surveys and Delphi studies instruments of the same kind—one simply more iterative than the other—or are they methodologically distinct in ways that matter for how we conduct, interpret, and critically appraise them?

The Delphi method is fundamentally distinct from cross-sectional surveys in its design and purpose. Delphi methodology is an iterative, structured process aimed at achieving consensus among knowledgeable participants or a panel of experts, through multiple rounds of questionnaires combined with controlled feedback. This iterative feedback allows participants to reconsider and refine their views toward a collective agreement, rather than merely recording individual opinion.

Despite a great focus on evidence-based medicine, Delphi studies are often used in healthcare, including critical care medicine, in areas where evidence is lacking, ambiguous, or heterogeneous (2). Delphi methods have been used for defining syndromes or constructs (3, 4), identifying knowledge gaps and research priorities (5), describing core outcome measures (6), and consensus recommendations for management of new or reemerging conditions (7, 8). Nevertheless, the consensus studies are based on judgment and experience of perspective studies and influenced by various methodological biases. In the absence of a universally accepted standards of conduct for Delphi studies, the methodological variations are common and influence the rigor, credibility and validly of the outcomes of these studies (9).

The Delphi questionnaire is the centerpiece of the exchange of information between the principal investigators and the participants (panelists). Although the questionnaire provides an opportunity to identify the knowledge gaps and research priorities, it is explicitly designed to generate agreement, not merely to measure preexisting opinion. Therefore, cross-sectional surveys and Delphi studies are methodologically distinct instruments, and understanding where their biases diverge is essential for better designing and critical appraisal (10).

## BIASES SPECIFIC TO DELPHI QUESTIONNAIRE

### Panel Composition Bias

**Figure 1.**
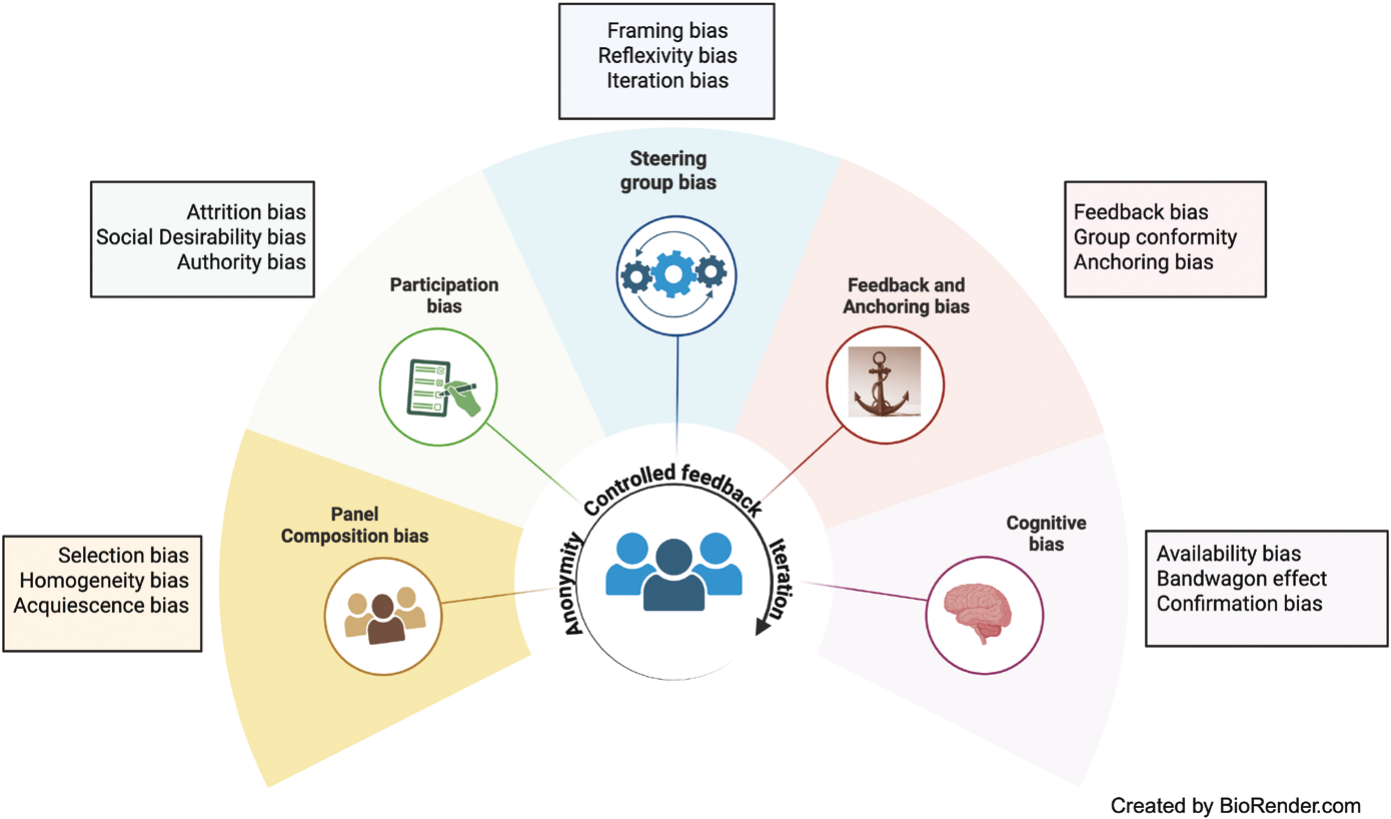
Key methodological and cognitive biases in Delphi questionnaire.

The selection of the expert panel is one of the most important methodological decisions in a Delphi study (**Figure 1**). Panelists (often referred to as experts) are rarely selected randomly and are typically nominated, purposively identified, or selected through a professional network. Biases include selection bias with geographical, institutional, or specialty overrepresentation and intentional exclusion of dissenting or minority viewpoints (acquiescence bias) (10). Homogeneity bias with common training and environment background and/or limited perspective produces artificial consensus (e.g., selection of panelists only from resource-rich settings) (11).

Panel composition bias is particularly detrimental in Delphi studies compared with cross-sectional surveys because the selected panelists play a significant role in influencing the final recommendations. Findings from cross-sectional surveys are primarily descriptive and do not have the same normative weight.

### Participation Bias

Delphi studies frequently experience attrition of panelists between rounds. Panelists who choose to withdraw may systematically differ from those who continue to participate; they may be more time-constrained, more dissenting, or less invested in the topic. However, the final consensus is derived solely from those who complete all rounds, and the sample becomes progressively narrower and potentially alters the strength of the consensus (attrition bias). Despite being a significant limitation, such changes are seldom reported with explicit transparency.

The identity of the panelist and the voting are anonymized to prevent peer influence. In a nonanonymous panel, junior or less prominent panelists may defer to a dominant or senior expert panelist, thereby compromising independent expert opinions. Even in anonymous panels, authority bias may persist if institutional affiliations are visible (12).

In Delphi studies, social desirability bias may arise when panelists tend to endorse statements that are perceived as professionally appropriate, culturally or ethically favorable, or aligned with prevailing clinical standards, even when their personal views differ (11).

### Steering Group Bias

The steering group play an important role in framing and modifying the statements of the Delphi questionnaire. The wording and selection of items are critical in Delphi studies, as they affect how statements are interpreted and how consensus is reached. Ambiguous, leading, or poorly worded statements may generate apparent consensus while masking uncertainty or steering responses in a particular direction.

The steering group decides the themes or domains, modifies the statements, and decides which items to include in subsequent Delphi rounds, thus introducing subjectivity. Methodological rules apply to both cross-sectional and Delphi questionnaire such as the use of neutral language and avoidance of double-barreled and double-negative statements. However, preconceived views within the steering group may unintentionally affect how statements are framed, revised, or retained across Delphi rounds, thereby influencing the emerging consensus. This form of reflexivity bias should be explicitly acknowledged and mitigated. The engagement of panelists through open-text comments in the initial Delphi rounds helps identify framing and reflexivity bias (13).

Finally, a fixed number of rounds limits the true impact of iteration and controlled feedback in the generation of consensus (iteration bias). Stability assessment between consecutive rounds helps to determine the shift in responses and the need for further iteration. The involvement of an independent methodologist and predefined rules for modification and the iterative process helps to mitigate steering group bias.

### Feedback and Anchoring Bias

Controlled feedback plays an important role in shifting panel perspectives. The approach used for summarization of feedback and presentation of round results can inadvertently steer opinions in a particular direction (feedback bias).

In-person meetings conducted to refine statements, resolve disagreements, or refine consensus statements are particularly vulnerable to group conformity bias. Social dynamics, peer influence, and the tendency toward group cohesion encourage panelists to adopt prevailing viewpoints rather than express dissent. Furthermore, even with anonymity, the direction of agreement from the majority of panelists can pressure panelists to shift toward consensus and suppress legitimate dissent (group conformity).

Finally, in later rounds of Delphi studies, anchoring bias may arise. Panelists rely disproportionately on initial statements, early-round results, preliminary consensus, or feedback provided between rounds rather than independently assessing the evidence or statement (14).

### Cognitive Bias

Cognitive bias refers to systematic and often unconscious deviations from objective reasoning that influence the perception, interpretation, and processing of information, as well as judgment and decision making (11). As a methodology that relies heavily on human judgment and interpretation, the Delphi process is inherently susceptible to cognitive bias (11, 12, 14).

Availability bias arises when panelists unintentionally and disproportionately weight recent clinical experiences, memorable events, or highly publicized cases, thereby influencing consensus formation independently of the underlying evidence base. Comments from outlier responses may suggest the presence of availability bias.

The bandwagon effect is typically observed when panelists shift their responses between rounds because of emerging evidence or consensus. Panelists align their views with the emerging consensus, artificially inflate agreement, and thereby, prematurely form consensus (15). Finally, preexisting beliefs or perceptions of panelists or the steering group led to confirmation bias in preferential supporting statements, interpretations, or modifications aligning with their thoughts. Cognitive biases operate largely outside conscious awareness; they may remain unrecognized unless actively anticipated and addressed.

A limitation shared by both cross-sectional survey research and Delphi methodology, and not addressed in the framework presented by Ramadurai et al (1), is the systematic privileging of consensus over dissensus. Cross-sectional surveys are typically evaluated by how well responses cluster; Delphi studies are considered successful when agreement is achieved. Yet in both cases, the variation or the dissensus may carry equal or greater scientific and clinical importance. In surveys, substantial heterogeneity in practice or opinion reveals the true state of a field. Consequently, summarizing findings solely through a modal response risks oversimplifying complex viewpoints and masking areas of uncertainty or disagreement. In Delphi studies, the items that persistently fail to reach consensus despite multiple rounds of iteration are not failures of the process; they are signals of genuine expert disagreement, often in precisely the domains where clinical uncertainty is greatest and where further research is most needed. Reporting and interpreting dissensus with the same rigor applied to consensus should be considered a methodological standard, not an afterthought.

## CONCLUSIONS

The methodological rigor of cross-sectional surveys and Delphi-based consensus studies is an important consideration. Our proposed taxonomy highlights multiple potential biases during panel selection, questionnaire development, and survey administration during Delphi rounds (**Table 1**). Rather than viewing dissensus as a methodological failure, future studies should explicitly report areas of persistent disagreement, as these often highlight uncertainties and research priorities. Greater transparency regarding panel selection and composition, questionnaire development, and biases is also essential to enhance trustworthiness and reproducibility. Finally, the incorporation of bias-appraisal frameworks into the design, conduct, reporting, and peer review of consensus studies may facilitate more rigorous evaluation of methodological quality.

**TABLE 1.** Methodological and Cognitive Biases in Delphi Studies

Type of Bias	At Which Level	
	Steering Group	Expert Panel
Selection bias	X	
Homogeneity	X	X
Acquiescence bias		X
Attrition bias		X
Social desirability bias		X
Authority bias		X
Framing	X	
Reflexivity	X	
Von Restorff		X
Feedback bias		X
Group conformity		X
Anchoring bias	X	X
Availability bias		X
Bandwagon effect		X
Confirmation bias	X	X
